# Synthesis, Functionalization, and Bioapplications of Two-Dimensional Boron Nitride Nanomaterials

**DOI:** 10.3389/fbioe.2019.00363

**Published:** 2019-12-10

**Authors:** Melis Emanet, Özlem Sen, Irem Çulha Taşkin, Mustafa Çulha

**Affiliations:** Department of Genetics and Bioengineering, Faculty of Engineering, Yeditepe University, Istanbul, Turkey

**Keywords:** two-dimensional boron nitride nanoparticles, synthesis, functionalization, biocompatibility, bioapplication

## Abstract

Two-dimensional boron nitride nanostructures (2D-BNNs) have been increasingly investigated for their applications in several scientific and technological areas. This considerable interest is due to their unique physicochemical properties, which include high hydrophobicity, heat and electrical insulation, resistance to oxidation, antioxidation capacity, thermal conductivity, high chemical stability, mechanical strength, and hydrogen storage capacity. They are also used as fillers, antibacterial agents, protective coating agents, lubricants, boron neutron capture therapy agents, nanocarriers for drug delivery, and for the receptor phase in chemosensors. The investigations for their use in medicine and biomedicine are very promising, including cancer therapy and wound healing. In this review, 2D-BNNs synthesis and their surface modification strategies, biocompatibility, and bioapplication studies are discussed. Finally, a perspective for the future use of these novel nanomaterials in the biomedical field is provided.

## Introduction

It is generally considered that boron nitride (BN)-based nanomaterials are not naturally formed, but the cubic crystalline boron nitrides (cBNs) are found in some rare mineral sites of Tibet and China, which suggests their natural occurrence (Du Frane et al., 2016; Yin et al., 2016). Today, most BN nanomaterials are synthesized in laboratories and are composed of an equal number of boron (B) and nitrogen (N) atoms that have specific conformations leading to different structure crystallinity (Pakdel et al., 2012). As the most stable form of BNs, hexagonal boron nitrides (hBNs) have strong covalent bonds between B-N atoms with a graphene-like structure. The 2D BN layers are held together through van der Waals interactions (Weng et al., 2016). On the other hand, in the rhomboedral (rBNs), diamond-like (cBNs), and wurzite boron nitrides (wBNs), B and N atoms are sp^3^ hybridized, binding to the neighboring BN_3_ tetrahedrons at different angles restraining the identical pattern of B and N atoms. The varying crystallinity in BN structures are obtained under different experimental conditions, such as temperature and pressure (Merlo et al., 2018). While hBNs and rBNs are produced at ambient pressure and at high temperature, wBNs form hBNs under high pressure at room temperature. cBNs are prepared from hBNs under high pressure at high temperature (Han, 2010).

BN nanomaterials are the structural analogs of graphene. The main difference between BN-based nanomaterials and their C counterparts is the nature of the bonds between the atoms. The bond C-C in carbon nanomaterials has a pure covalent character, while B-N bonds present a partially ionic character due to the e^−^ pairs in sp^2^ hybridized B-N. The e^−^ pairs are more confined to N atoms owing to their high electronegativity in BN-based nanomaterials that strongly affect mechanical, optical, and electronic properties (Arenal and Lopez-Bezanilla, 2015; Jiang et al., 2015). Therefore, BN-based nanomaterials can improve thermal conductivity (Chang et al., 2005) and the mechanical (Sen and Çulha, 2015; Emanet et al., 2016) and antioxidant (Chen et al., 2004; Li et al., 2018) properties of several composites. High thermal conductivity and improved mechanical properties, including high tensile strength and elasticity, which BN-based nanomaterials can provide, are in high demand, for example for the construction of tissue-mimicking biomaterials used in transplantation (Jo et al., 2013). The finding that BN-based nanomaterials might have piezoelectric properties may allow for the generation of novel biomaterials used in tissue-engineering processing (Merlo et al., 2018). Another important property includes making BN-based nanomaterials more favorable for additives, as compared to their C counterparts, is their electrical insulation.

In recent years, BN-based nanomaterials have grasped the attention of researchers for their possible use in medical applications due to their good biocompatibility (Chen et al., 2009; Salvetti et al., 2015; Li et al., 2017) and high chemical and mechanical stability (Lahiri et al., 2011; Liu et al., 2015). In addition, their cellular internalization allowed the researchers to investigate their possible use as drug and gene carriers (Horvath et al., 2011). The therapeutic effects of boron compounds in cancer treatment also made the BN-based nanomaterials interesting structures as controlled release boron sources (Li et al., 2017). They have also been widely used in cosmetics, lubricants, insulators, and microwave-transparent materials (Jiang et al., 2015). cBNs are also called white diamonds because of their high Young's modulus compared to diamonds. They are used to cut many industrial ferrous materials, and they do not react with the related alloys, as is the case with diamonds (Han, 2010).

**Figure 1:**
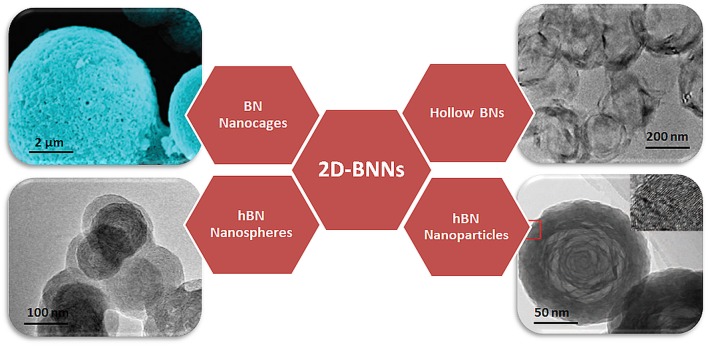
Schematical representation of several physical forms of BN nanospheres. Reproduced with Permission from Tian et al. (2013) and Zhang et al. (2012).

One-dimensional (1D) nanotubes and nanoribbons, two-dimensional (2D) nanospheres and nanosheets, and three-dimensional (3D) nanoporous BNs are typical examples of BN nanomaterials (Jiang et al., 2015). Among these nanomaterials, low dimensional nanomaterials show quantum confinement and interfacial effects compared to 3D nanomaterials. There is therefore a significant effort to use these nanomaterials with unusual physicochemical properties in novel applications (Pakdel et al., 2012). Moreover, several physical forms of the 2D nanospheres, including nanocages, nanococoons, and hollow BNs, have been synthesized, as shown in Figure 1, thereby providing an opportunity to choose a suitable form of 2D nanostructure for the desired applications.

In this review, synthesis methods of 2D-BNNs will be discussed first, and then their functionalization, biocompatibility, and bioapplications in drug delivery, composites, cosmetics, therapeutics, and pharmaceuticals will be discussed.

## Synthesis of 2D-BNNs

The synthesis of different forms of 2D-BNNs is widely investigated in the literature, and the most relevant ones to the subject of this review are discussed here. Table 1 shows some of the methods and experimental conditions used to synthesize 2D-BNNs. As seen, several B and N precursors were used to obtain BN-based nanomaterials with different sizes and shapes.

**Table 1:** Reaction conditions and methods to produce 2D-BNNs.

**Precursors**	**Environmental conditions**	**Catalyst**	**Methods**	**Purifying**	**Product**	**References**
Boron-rich conducting electrodes	N_2_ gas	Nickel, cobalt	Plasma-arc discharge	None	hBN Nanococoons	Cuming and Zettl, 2000
(B(OMe)_3_)	NH_3_ gas, 800–1,500°C	None	CVD	None	BN spheres	Wood et al., 2006; Tang et al., 2008
(B(OMe)_3_)	NH_3_ and Ar gas, 800–1,500°C	None	CVD	None	Hollow BNs	Li et al., 2017
NH_4_BF_4_ and NaN_3_	N_2_ gas, 250°C, 450 MPa	S	Ball milling	(HCl), C_6_H_6_, ddH_2_O	hBN	Lian et al., 2010
H_3_BO_3_, C_3_H_6_N_6_	NH_3_ gas, 1600°C	None	CVD	None	BN	Ansaloni and Sousa, 2013
Carbon nanocages, B_2_O_3_	NH_3_ gas, 1350-1450°C	None	Elemental substitution reaction	None	BN and BCN nanocages	Suryavanshi et al., 2014
Boron powder	NH3 gas	Fes/Fe_2_O_3_	TCVD	None	BN nanosheets	Ansaloni and Sousa, 2013
H_3_BO_3_, Colemanite, B_2_O_3_	NH_3_ gas, 1300°C	None	CVD	None	hBN	Sen et al., 2018

2D-BN nanococoons are produced through a synthesis approach typical of that followed for boron nitride nanotubes (BNNTs) using an arc discharge method already established in 2000 (Cuming and Zettl, 2000). Although this method is the easiest bulk quantity carbon nanotube production method, it was not efficient to be used for production of BN-based nanomaterials because of their non-conductive nature. To establish electrical conductivity, the use of a metal was reported in the literature (Chopra et al., 1995). However, it resulted in accumulation of high amounts of metal impurities in the synthesis environment. In the case of the 2D-BN nanococoons mentioned above, 99% elemental boron as a boron precursor, with 1% nickel and cobalt acting as a catalyst, was brought into close contact with electrodes. The synthesis procedure generated clustered nanocrystals individually coated with multi-layer graphite-like BNs. The authors managed to remove the interiors of the nanocrystal from the BN cage structures to obtain hollow BN “nanococoons.”

BN spheres are one of the commonly reported structures, and the chemical vapor deposition (CVD) is a golden-standard synthesis method. For example, hexagonal BN spheres were synthesized through a two-step procedure using CVD (Wood et al., 2006). In the first step, B-N-O particles were synthesized from trimethyl borate (B(OMe)_3_) and ammonia precursors; then, oxygen atoms were removed from spheres by heating them under an ammonia atmosphere at high temperature. Tang et al. claimed that the (OMe)_3_BNH_3_ complex was produced in place of removing oxygen atoms from the structure at the second step (Tang et al., 2008). Another intermediate phase was therefore introduced to produce BN spheres. The BN ceramics were formed using dehydrogenative hydrolysis from BH_3_NH_3_ to eliminate hydrogen. At the end, B-O impurities generated the nuclear center of BN spheres with a size range of 50–400 nm (Tang et al., 2008). In a further study, argon (Ar) gas was exploited instead of NH_3_, allowing B-O evaporation from the inner part of nanospheres during the second phase of the synthesis to obtain hollow BN nanomaterials in a hexagonal lattice structure (Li et al., 2017).

The synthesis of nearly monodisperse 2D-hexagonal boron nitride nanoparticles (2D-BNNs) was achieved via a modified solid-state metathesis reaction route (Lian et al., 2010). In the study, sulfur was used as catalyst to produce 2D-BNNs at a relatively low temperature of 250°C. With this information on hand, the synthesis was attempted using ammonium borofluoride (NH_4_BF_4_), with sodium azide (NaN_3_) used as precursors and sulfur as a catalyst. The mixture was ball milled, and then pressed at 250°C for 20 h. Finally, the product was washed with hydrochloric acid (HCl), benzene (C_6_H_6_), and deionized water to remove iron, sulfur, and water-soluble salt impurities, respectively. Nearly monodisperse 2D-BNNs, in the size range of 35–45 nm, were obtained progressing the synthesis efforts toward a high yielding low-energy state.

**Figure 2:**
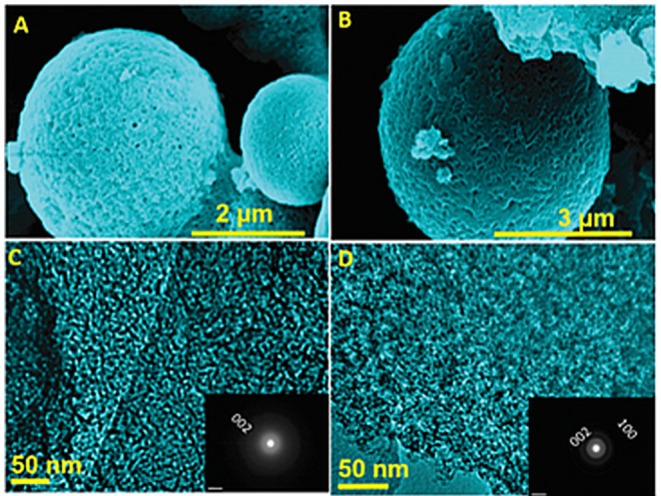
HRSEM and HRTEM images of 2D-BNN and 2D-BCNN nanocages (**A,C**: 2D-BNN; **B,D**: 2D-BCNN). Reproduced with Permission from Suryavanshi et al. (2014).

The ceramic structure of 2D-BNNs has become an interesting material used in cosmetic formulations to provide better lubricity and other uses, such as blocking UV radiation. Thus, their synthesis in large quantities is necessary. Addressing this issue, a two-step synthesis procedure was reported (Ansaloni and Sousa, 2013). In this procedure, first, boric acid (H_3_BO_3_) and melamine (C_3_H_6_N_6_) solutions were prepared and mixed for 48 h. Then, their precipitate was filtered and dried at 37°C. The obtained product, B_4_N_3_O_2_H (precursor of 2D-BNNs), was first heated to 500°C, and, then, it was placed in an aluminum boat to heat at 1,600°C under ammonia gas flow for 2 h. This procedure yielded large amounts of hBN-based 2D-BNNs, and their infrared light absorption ability was very good at making these materials powerful additives for sunscreen application. The synthesis of spherical BN and BCNs was also reported. The synthesis was accomplished via an elemental substitution reaction, as shown in Figure 2, where carbon nanocages were used as a template, and boron trioxide (B_2_O_3_) was used as a boron source (Suryavanshi et al., 2014). They were mixed and heated under an NH_3_ gas flow for a substitution reaction. Microspheres with a size range of 1–3 μm were formed with interconnected holes, suggesting the elemental substitution of B and N atoms in place of C atoms. The obtained product had a highly crystalline wall structure and uniform pore size distribution, further confirming the elemental substitution process.

Considering their biocompatibility and low cytotoxicity, BN-based nanomaterials can also be used in biological applications, including imaging. In one report, it was shown that hBN-patterned 2D-BN nanosheets had luminescent properties (Ansaloni and Sousa, 2013). They were synthesized from boron powders with NH_3_ gas as precursors and FeS/Fe_2_O_3_ as catalysts via a thermal chemical vapor deposition (TCVD) method, and they were well-characterized, using several spectroscopic and imaging techniques to ensure the structural elucidation of the material (Ansaloni and Sousa, 2013). We also successfully synthesized hBNs with a one-step synthesis method using H_3_BO_3_, colemanite, or B_2_O_3_, as boron precursors (Sen et al., 2018). The synthesis was performed via the CVD method under ammonia gas flow at 1,300°C for 2 h. In this synthesis method, we did not use a catalyst, creating the opportunity to prepare completely pure hBNs. We also found that the boron source had an influence on their crystallinity, stability, and biodegradability in suspensions mimicking oxidative and hydrolytic degradation environments. We have concluded that an appropriate boron precursor should have been chosen depending on the target application when the crystallinity of the product is critical.

Based on the synthesis methods reported to date, it is clear that the synthesis parameters, including precursors, temperature, and pressure, are acutely important to the structure of 2D-BNNs and have a significant effect on their crystal and morphological structures. Thus, the synthesis method vs. the target application should be carefully considered. At the moment, we are not at the point where the synthesis method and the structural relationship are clearly established. More work is needed to address this point for the full benefits of these novel structures. Although a certain level of success has been achieved, a significant effort to develop a universal approach satisfying desired product quality is necessary.

## Functionalization of 2D-BNNs

### Chemical Functionalization of 2D-BNNs

The functionalization of 2D-BNNs is specifically aimed at improving their applicability in biomedical applications through several routes. 2D-BNNs are composed of an equal number of sp^2^ hybridized, covalently linked B and N atoms, which generate a highly stable hexagonal BN network. Differences in electronegativity in B and N atoms result in also making the structure interesting from a functionalization point of view. Electron pairs in their sp^2^ hybridization are closer to N atoms due to their higher electronegativity making B atoms more electron deficient and favorable for covalent functionalization (Ikuno et al., 2007). Both N and B atoms are targeted for functionalization at the layer edges or defects. Either a hydroxyl group is attached to B or N atoms are converted to -NH_2_ groups.

A summary of recent reports demonstrating the functionalization of 2D-BNNs and their final products is provided in Table 2. Despite the promising progress in the functionalization strategies, the yield is still lower than the desired target. This can perhaps be explained with the highly stable and inert chemical structure of 2D-BNNs.

**Table 2:** Several functionalization approaches of 2D-BNNs.

**Precursors**	**Chemicals**	**Method**	**Purification**	**Product**	**References**
BN	ddH_2_O	Sonication	None	BN-OH	Lin et al., 2011
BN	Alkoxyl groups	Solution phase oxygen radical functionalization	Hydrolytic defunctionalization	BN-OH	Sainsbury et al., 2012
BN	Sodium hydroxide	Ball milling method	None	BN-OH	Lee et al., 2015
BN	N_3_C_4_	Reverse reaction (elemental substitution reaction)	None	BN-OH	Weng et al., 2014
BN	PEG	Exfoliation	None	BN-NH_2_	Lin et al., 2009
BN	Urea	Ball milling method	None	BN-NH_2_	Lei et al., 2015

In one hydroxylation study, a simple sonication in deionized water was used to generate -OH on B atoms, which was named as a “clean” method (Lin et al., 2011). The formation of hydroxyl groups was confirmed from the FT-IR spectra through the peaks that appeared at around 3,414 cm^−1^. In another study, the formation of -OH groups on B atoms was attempted in the solution phase by forming oxygen radicals (Sainsbury et al., 2012). This procedure involves two steps; first, chemically grafting alkoxyl groups on 2D-BNNs, followed by the hydrolytic de-functionalization of the groups, using acidic solutions to obtain the hydroxyl groups. From the TGA analysis, it was found that about 4 wt% of boron atoms in the lattice were functionalized by taking the tert-butoxy groups into account. Along a BN sheet edge, the presence of four boron atoms at every nanometer was predicted, which means that sheet-edge boron atoms are 0.044% of the total atoms in the nanosheets. Since TGA analysis indicates that about 4% of boron atoms are functionalized, about two orders of magnitude difference suggest the functionalization of the center atoms of the nanosheets through the proposed oxygen radical assertion functionalization mechanism. Lee et al. proposed a scalable exfoliation process for the hydroxylation of 2D-BNNs using a ball-milling method with sodium hydroxide (Lee et al., 2015). This sodium hydroxide-assisted ball-milling process generated a higher yield (18%) of 2D-BNN hydroxylation, and they were stably dispersed in various aqueous environments that were essential for biomedical applications. In a new and interesting method, BNs with high hydroxylation degrees using thermal substitution of C atoms with boric acid substructures in graphitic carbon nitrides (g-C_3_N_4_) were prepared. The synthesized BN(OH)x (x = 0.6–0.9) was highly water soluble and porous. The suspension was prepared by dispersing it as high as 2.0 mg mL^−1^ in water, and it was stable and highly transparent (Weng et al., 2014).

Generating amine groups from N atoms in the structure of 2D-BNNs was first studied by Lin et al. by inducing the exfoliation of 2D-BNNs into some layers or monolayers to obtain electron-deficient B atoms for amine group formation through Lewis-base interactions (Lin et al., 2009). The synthesized amine group-functionalized 2D-BNNs were significantly soluble in water and common organic solvents. In another study, amine functionalization was performed by urea-assisted exfoliation of the 2D-BNNs using, once again, mechano-chemical reactions. The 2D-BNNs and urea were ball-milled under nitrogen atmosphere to generate amino groups (Lei et al., 2015).

## Biocompatibility of 2D-BNNs

**Figure 3:**
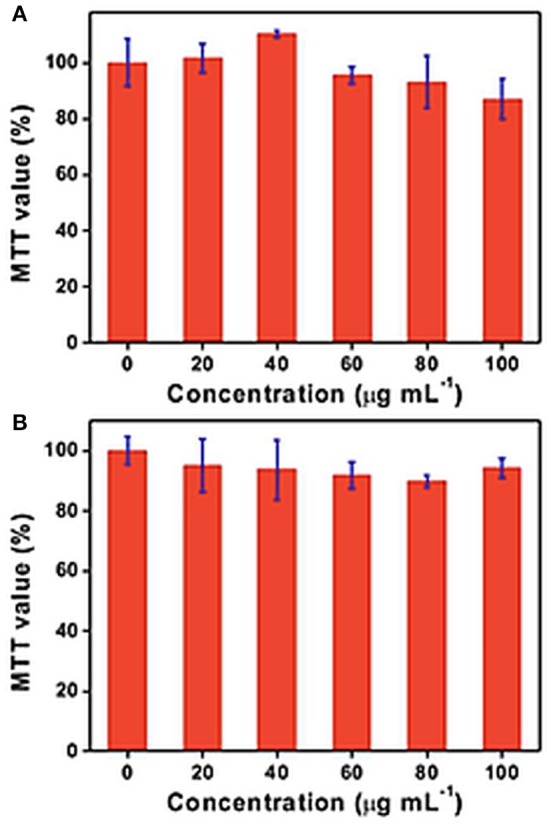
MTT assays on **(A)** HEK-293T and **(B)** CHO cell cultures incubated for 48 h with different concentrations of h-BN nanoplates. Results are presented as mean value ± standard error; *n* = 3 for the MTT assays. Republished with the permission of [Royal Society of Chemistry], from Lu et al. (2016); permission conveyed through Copyright Clearance Center, Inc.

Biocompatibility is a crucial step that should be clarified in order to identify any potential adverse effects before using nanomaterials can be used for biomedical applications. Although 2D-BNNs have been employed in biomedical applications, there is no clear consensus for their biocompatibility so far. In some studies, it has been found that the toxicity of the hBNs is dependent on cell type, dosage, and aspect ratio. In an attempt to evaluate the biocompatibility of hBNs, Lu et al. reported that the concentrations of 2D-BNNs up to 100 μg/mL with lateral size dimensions of 30–60 nm did not significantly affect the viability of HEK-293T and Chinese hamster ovary (CHO) cell lines (Lu et al., 2016). The findings were based on MTT [3-(4,5-dimethylthiazol-2-yl)-2,5-diphenyltetrazolium bromide, a tetrazole] colorimetric assay after 48 h incubation as shown in Figure 3. Furthermore, 2D-BNNs did not increase the apoptotic rate of both cell lines suggesting the safe use of 2D-BNNs in living systems. In another study, 2D-BNNs with average particle size of 121 nm were tested using Madin-Darby Canine Kidney (MDCK) and human normal skin fibroblast (CRL 2120) cell lines (Kivanç et al., 2018). The cell viability was assessed again using MTT, sulforhodamine B (SRB), and PicoGreen assays. The results indicated that the 2D-BNNs could be used up to 100 μg/mL on both cell lines without showing any cytotoxicity. The study concluded that 2D-BNNs could be considered potentially safe for oral care products (Kivanç et al., 2018).

**Figure 4:**
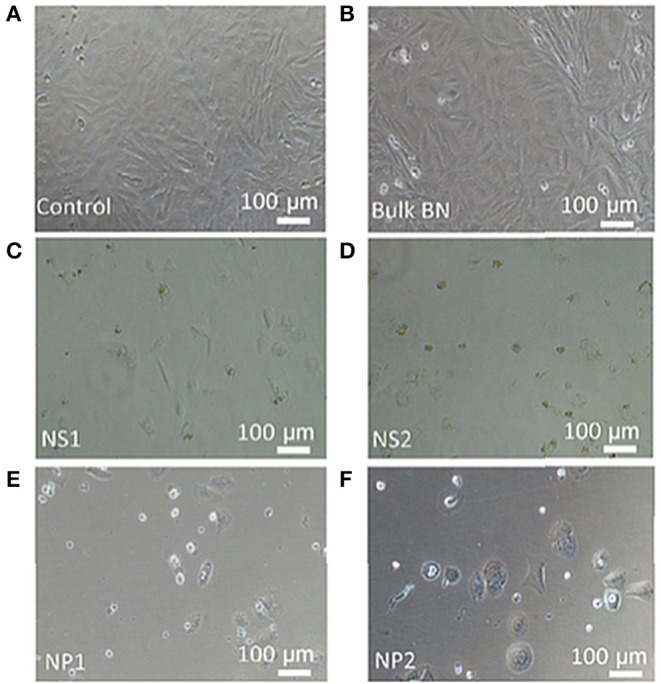
Bright-field microscopy images of SaOS2 cells cultured in the presence of **(A)** standard culture medium (control), **(B)** bulk BN, **(C)** nanosheet NS1, **(D)** nanosheet NS2, **(E)** nanoparticle NP1, and **(F)** nanoparticle NP2. Reproduced with Permission from Mateti et al. (2018).

Li et al. investigated 2D-BNNs with lateral size dimensions of 50–60 nm on androgen-sensitive LNCaP and androgen-independent DU145 prostate cancer cell lines up to 25 μg/mL with 3 and 6 days of incubations (Li et al., 2017). The cell viability was assessed using WST-8, annexin V-FITC/propidium iodide (PI), and LDH assays. It was found that 2D-BNNs crystallinity affected the B release, and this consequently decreased the cell viability at increasing concentrations and incubation times, also inducing apoptosis. The results suggested that 2D-BNNs could be used as a novel therapeutic agent in prostate cancer treatment. Further, Mateti et al. performed MTS and 2,2-diphenyl-1-picrylhydrazyl (DPPH)-free radical assays to evaluate the cytotoxicity and biocompatibility of 2D-BNNs (Mateti et al., 2018). Micrometer-sized 2D-BNNs with a concentration of 1 mg/mL showed that they are biocompatible on the SaOS2 cell line (a human osteosarcoma cell line). However, 2D-BNNs with a concentration of 1 mg/mL and a diameter smaller than 1 μm showed lesser biocompatibility and produced ROS. Bright-field microscopy images of SaOS2 cells also supported the findings obtained from biocompatibility studies, as shown in Figure 4. Figure 4C shows that the diameter of 2D-BNNs is around 1 μm, and the thickness is around 100 nm (referring to as NS1). Figure 4D shows smaller 2D-BNNs with a diameter of 100 nm and a thickness of ~3 nm (referring to NS2). Figure 4E shows the NP1, which has a diameter in 110 nm, and the smaller nanoparticles that have diameters in the range of 10–40 nm (referred to as NP2) were shown in Figure 4F. In conclusion, this study revealed that the biocompatibility of 2D-BNNs depends on structure, size, and shape, which was similar to the findings of the biocompatibility studies in the case of all other nanomaterials. Furthermore, the boron radicals that occur in the edges of the BN nanosheets could be the cause of ROS leading to cell death.

In another study, the cell viability of hBNs and water-soluble hydroxyl-functionalized hBNs (hBN-OH) that have a size range of 50–100 nm in diameter were tested on the KB (human cervix carcinoma) cells up to 500 μg/mL concentrations using an MTT assay. Even at the higher concentrations, no toxicity was observed after 24 h incubation, thereby suggesting the use of hBN-OH for imaging and *in vitro* detection applications (Nurunnabi et al., 2016).

There is a limited number of biocompatibility studies in the literature so far, and all are *in vitro*. The results of these reports are summarized in Table 3. In all of the studies, the 2D-BNNs are claimed to be non-toxic, but their biocompatibility clearly depends on several parameters, including cell type, dose, type of dispersion surfactant, and their lateral size dimensions. In summary, we can conclude that 2D-BNNs have no significant adverse effects on the cell proliferation, metabolism, and viability but further *in vivo* studies should be performed to reach more concrete conclusions.

**Table 3:** Cytocompatibility of 2D-BNNs on culture cell lines.

**Dispersion agent**	**Cell lines**	**Toxicity assays**	**Incubation time**	**Results**	**References**
None	HEK-293T and CHO	MTT and annexin V-FITC/PI	2 days	Nontoxic ≤ 100 μg/mL, no apoptosis	Lu et al., 2016
None	CRL 2120 and MDCK	MTT, SRB colorimetric, PicoGreen	1 or 2 days	Nontoxic ≤ 100 μg/mL for both cell lines	Kivanç et al., 2018
None	LNCaP and DU145	WST-8, annexin V-FITC/PI, LDH	3 or 6 days	Time-dependent toxicity, induce apoptosis	Li et al., 2017
None	SaOS_2_	MTS, DPPH assay	7 days	Size-dependent toxicity and ROS production	Mateti et al., 2018
None and hBN-OH	KB	MTT	1 days	Nontoxic ≤ 500 μg/mL	Nurunnabi et al., 2016

## Bioapplications of 2D-BNNs

### 2D-BNNs in Drug Delivery Applications

A successful drug carrier requires the fulfillment of certain constraints, including low toxicity, flexibility, and stability in biological environments, and the successful carrying and release of drug molecules into cells at the target tissue. Numerous nanomaterials including polymeric and non-polymeric ones have been investigated for their drug carrier potentials since nanomedicine concept has been introduced. As the nature of BN based nanomaterials started to be better understood, as shown in Table 4, they have also been gained interest for drug delivery applications. Here, we will discuss their potential use as drug carriers in the light of reported studies so far after a brief introduction to the challenges in drug delivery.

**Table 4:** Bioapplications of 2D-BNNs.

**2D-BN Nanomaterial**	**Application**	**Result**	**References**
BNNS/CpG oligonucleotides	Drug delivery, immunotherapy	Show great capacity to stimulate IL6 and TNF-α production, increased cytokine production	Zhang et al., 2012
Chitosan coated BNNS/CpG oligonucleotides	Drug delivery, immunostimulatory	Higher immunostimulatory effects increasing cytokines (IL-6 and TNF-α) in TLR9 cells even more than positive control (lipofectamine-CpGs)	Zhang et al., 2013
BNNPs-Dox	Drug delivery	Efficient cellular internalization of BNNP-Dox and serious Dox release	Sukhorukova et al., 2015
BNNS-FA/Dox	Drug delivery	pH dependent Dox release, greater cytotoxic effect on HeLa cells	Feng et al., 2016
FA-Cyst-Ag/BNNPs	Drug delivery	Positive effects on cancer cell targeting	Permyakova et al., 2017
Dox-hBN	Drug delivery	pH dependent Dox release	Emanet et al., 2017
AuNPs-BNNs	Drug delivery	Attractive materials for cancer drug delivery and photodynamic therapy	Jedrzejczak-Silicka et al., 2018
2D-BN nanoparticles	Drug delivery, spectroscopic marker	Tumor cell perturbation	Gnatyuk et al., 2018
DOX@PAH-cit–BNNS	Drug delivery	Decreased cell viability in both MCF-7 and HeLa cells more than free DOX	Feng et al., 2018
BNNP/PPF	Bone tissue engineering	Enhanced mechanical strength and adsorption of collagen I protein, improved ECM deposition, cell attachment and spreading for bone grafts	Farshid et al., 2015
BNNSs/AKM scaffolds	Bone tissue engineering	Increase compressive strength and fracture toughness	Shuai et al., 2015
hBNs/gelatin ESM	Orthopedic applications, tissue engineering,	Biocompatible and biodegradable scaffolds for orthopedic applications	Nagarajan et al., 2017
OH-BNNS/PVA	Drug delivery, artificial cartilages	Controllable reinforcements in both mechanical and thermal responses	Jing et al., 2017
PEEK/hBN	Nanocomposite	Improved mechanical and thermo mechanical properties	Liu et al., 2015
hBNs-impregnated silane	Bioimplant	5-fold improvement in the corrosion resistance in simulated human body fluid even after 96 h	Al-Saadi et al., 2017
Hollow BNs	Prostate cancer treatment	More suppressive effects on tumors as compared to the PTX drugs	Li et al., 2017
hBNs	Lubricant	Better lubrication and maximum performance in LPEF	Turkoglu et al., 2005
hBNs	Lubricant	Most effective lubricant based on LFEP-lubricant concentration profile, higher concentration of hBN caused lower mechanical properties	Ugurlu and Turkoglu, 2008
BN nanoparticles	Cosmetic	Improved skin appearance by not only blurring lines and wrinkles but also providing coverage of age spots, blemishes and discolorations	Butts et al., 2007
BN fillers	Cosmetic	Enhanced filling properties and illusion about smoothness of skin by hiding the wrinkles	Newman et al., 2015
BN nanoparticles/coated with amino acids and mineral oils	Cosmetic	Improved ingredients of cosmetic product	Koshida et al., 2019

All nanocarriers should pass through a number of biological barriers at organism and cellular levels. Therefore, an ideal carrier should have certain properties including stability, flexibility, size and shape, low toxicity and biodegradability as mentioned above for their full consideration as a nanocarrier. At the organism level, a nanocarrier should be able to carry a drug into the target region without drug leakage from the structure. Once the drug-loaded carrier reaches the target tissue, passing through the barriers, including the reticuloendothelial system (RES), at an organism level, it should trigger endocytosis pathways for cellular internalization at a cellular level, and it should then release its cargo into cells (Soma et al., 1999). Considering the properties of an ideal nanocarrier, 2D-BNNs can be good candidates through several of their unique properties, including flexibility, stability, and biocompatibility.

It is now clear that the size of the nanoparticles should be small enough to travel in the narrow blood vessels and penetrate into tumor tissue while also being large enough so as not to fenestrate into the endothelial lining (Champion et al., 2007). Therefore, spherical 2D-BNNs, in the size range of 20–200 nm, are good candidates to be used as carriers due to their biocompatible nature that allows them to interact with the hydrophobic sites of drug molecules (Emanet et al., 2017). Moreover, 2D-BNNs can be synthesized in different sizes that allow for the investigation of BNs for optimum drug loading and delivery performance.

2D-BNNs were first used as carriers to improve immunostimulator vaccines, CpG oligonucleotides, allergy immunotherapies, and cancer and infection treatment (Zhang et al., 2012). The CpG oligonucleotides are similar to DNA motifs in bacteria that stimulate a similar immune response in the body (Pisetsky, 1996). The interaction of CpG oligonucleotides with TLR9 antigen-presenting cells activates two distinct signaling pathways, in which two transcription factors, NFκB and IRF-7, are translocated to the nucleus, thereby leading to the induction of genes encoding pro-inflammatory cytokines [interleukin (IL)-6 and IL-12] and/or type I interferons (interferon-α) (Wagner, 2002). In the study, 2D-BN nanospheres interacted with CpG oligonucleotides, and they were investigated in immunotherapy applications (Zhang et al., 2012). Due to the electrostatic repulsion between negatively charged CpG oligonucleotides and 2D-BNNs, the 2D-BNNs were first coated with peptides composed of several amino acid combinations. A 12 amino acid-long peptide with a tyrosine residue (Y) at the 8th position from the N-terminus played a crucial role in the affinity to 2D-BNNs. The peptide-bound 2D-BNNs were internalized into the cells successfully and showed no toxicity to the peripheral blood mononuclear cells. Using the peptide as a cross-linker, the CpG oligonucleotide-binding efficiency was, to 2D-BNNs, 5-fold higher than the directly binding efficiency of CpG on 2D-BNNs. Moreover, the 2D-BNNs-loaded peptide-CpG conjugates had great capacity to stimulate IL6 and TNF-α production from cells. The increased cytokine production capacity could be attributed to the higher binding capacity and strong interaction of CpG oligonucleotides to 2D-BNNs.

**Figure 5:**
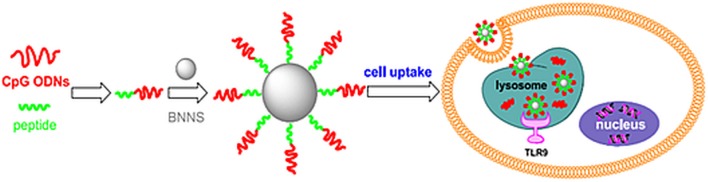
Using a boron nitride nanosphere (BNNS)-binding peptide as a linker molecule, BNNS are able to efficiently deliver immunostimulatory CpG ODNs into cells and significantly enhance the immune response. Reproduced with Permission from Zhang et al. (2012).

In a follow-up study, CpG oligonucleotides and 2D-BNNs interactions were studied after coating 2D-BNNs with positively charged chitosan to provide electrostatic attractions, as schematically shown in Figure 5 (Zhang et al., 2013). The chitosan was chosen as low (60–120 kDa), medium (110–150 kDa), and high (140–220 kDa) molecular weights to compare the CpG oligonucleotide-binding yield to chitosan-BNNs and their immunostimulatory effects. The results indicated that the CpG oligonucleotide-binding yield was higher to the high molecular weight chitosan-coated 2D-BNNs as well as their increased cellular uptake due to the high positive charge of the structure. However, the low molecular weight chitosan-BNNs interacted with CpG created a higher immunostimulatory effect by increasing the cytokines (IL-6 and TNF-α) in TLR9 cells even more than the positive control (lipofectamine-CpGs) (Zhang et al., 2013).

In another study, the porous structure of 2D-BN nanospheres was utilized as a nanocontainer for Dox, a commonly used chemotherapeutic drug (Sukhorukova et al., 2015). The loaded Dox was stable at neutral pHs (pH 7.4) and Dox was significantly released from 2D-BNNs at low pHs (pH 4.5–5.5), suggesting that the release in the cell will be effective. Efficient cellular internalization and Dox release are two important factors qualifying the structure as a potential carrier. Moreover, the cell viability tests showed the 2D-BNN-Dox structures were highly toxic for neoplastic IAR-6-1 cancer cells (Sukhorukova et al., 2015).

Feng et al. developed a targeted drug delivery system using folic acid (FA)-conjugated 2D-BN nanospheres (Feng et al., 2016). The FA molecules were grafted on 2D-BNNs via an esterification reaction. The biocompatibility results indicated that the 2D-BNNs-FA structures were not significantly cytotoxic on HeLa cells up to 100 μg/mL. The 2D-BNNs-FA structure was loaded with Dox. The 2D-BNNs-FA/Dox complexes also had high stability at pH 7.4 while also exhibiting a high Dox release performance at low pH values (pH 5.0). Besides, the cytotoxicity results indicated that the 2D-BNNs-FA/Dox structures have a greater cytotoxic effect on HeLa cells due to the overexpressed FA receptors. Therefore, the 2D-BNNs-FA/DOX complexes were recognized as a high performance Dox internalization agent as chemotherapeutic drug for HeLa cells (Feng et al., 2016). In a follow-up study, FA molecules were conjugated to the 2D-BNNs with a three-step procedure that included FA pre-activation by N,N′-dicyclohexylcarbodiimide (DCC), BNN modification with AgNPs to provide coupling of FA to BNNPs, and their further modification with L-cysteine to provide -NH_2_ ends (Permyakova et al., 2017). The final product was an FA-bound 2D-BNN structure (FA-Cyst-Ag/BNNs). The carboxyl-groups of FA were preactivated with DCC. Cyst, possessing a pair of cystine electrons (R–S–, Lewis base), donated its electron to the Ag+ (Lewis acid), leading to the formation of a zero-oxidation state of Ag atoms that resulted in chemically bonded Ag–S bonds via hydrocarbon chains of the cysteine. In the last stage, FA was grafted to the surface of BNNPs under a condensation reaction between amino groups of Cyst-Ag/BNNPs and carboxyl groups of FA using DCC. The study found that conjugating FA to the structure did not alter the targeting efficiency of FA.

In our group, hBNs were investigated as drug carriers by loading them with Dox (Emanet et al., 2017). The nature of the interaction between hBNs and Dox was non-covalent through the aromatic rings of Dox. The optimal loading was achieved at neutral and basic pH values with respect to acidic pH values. The Dox release studies indicated that the Dox-hBN conjugates were highly stable at around pH 7, while the Dox release from 2D-BNNs was triggered at low pH (pH 4).

**Figure 6:**
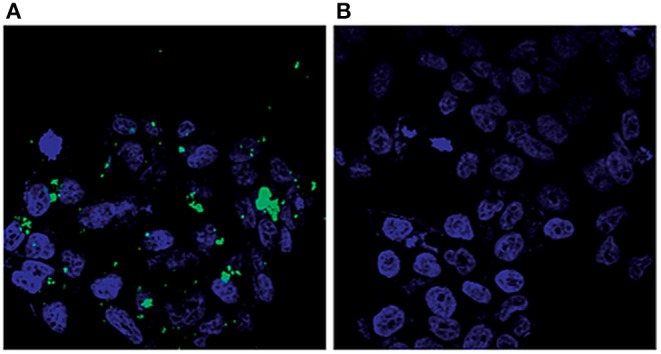
Visualization of LNCaP cells by confocal microscopy: **(A)** cells treated with modified 2D-BN nanoparticles and **(B)** reference cells (green fluorescence: FITC, blue fluorescence: Hoechst 33258). (Gnatyuk et al., 2018): Published by The Royal Society of Chemistry.

The spectral changes of the cell membrane and cytoplasm, along with the cellular internalization of Dox-loaded 2D-BNNs, were used as a marker to monitor the cellular uptake of a nanocarrier (Gnatyuk et al., 2018). The luminescence of the 2D-BNNs-Dox was monitored using confocal microscopy to visualize the time-dependent cellular localization, as shown in Figure 6. The anticancer efficiency of the structure was also investigated using IR and Raman spectroscopy. The luminescent effect of the 2D-BNNs was revealed in the LNCaP cells, especially when the cells were seeded on a gold substrate. The cellular internalization was completed at the first hour of incubation and realized from the strong spectral changes in Raman spectra obtained from the membrane lipid of the cells. Then, the normalized spectral results obtained from the second hour to 10 h of study claimed the localization of the 2D-BNN in cytoplasm until nuclear internalization was completed after 10 h of incubation.

Silicka et al. investigated the drug delivery performance of gold nanoparticle-functionalized hexagonal-based 2D-BNNs (AuNP-BNNs) on L929 and MCF-7 cell lines (Jedrzejczak-Silicka et al., 2018). They found that the mitochondrial and lysosomal activities of the cells were significantly reduced at long incubation times of 48 and 72 h. They concluded that that conjugates of 2D-BNNs with AuNPs are promising materials for cancer drug delivery and are photodynamic. Feng et al. reported the development of a highly pH-responsive carrier structures using 2D-BN nanospheres for efficient drug release inside tumor cells (Feng et al., 2018). In the study, 2D-BNNs were functionalized with charge-reversal poly (allylamine hydrochloride)-citraconic anhydride (PAH-cit) following the hydroxylation and amino group modification of the 2D-BNNs. Then, the developed PAH-cit-BNNs was loaded with Dox molecules, which was 8-fold more efficient than loading free 2D-BNNs. Moreover, the DOX@PAH-cit–BNNs complexes caused a serious cell viability decrease (around 20%) in both MCF-7 and HeLa cancer cells of more than free DOX (around 40–50%) and DOX@PAH-cit (around 60–70%) structures (Feng et al., 2018).

Thus, far, all studies have aimed at understanding the potential of this novel material at a cellular level. In general, all studies found that 2D-BNNs have low cytotoxicity or are not toxic at all. The other interesting finding was the pH-dependent drug binding and release. The optimal loading was achieved at neutral and basic pHs, while low pH triggered the increased release of the drug from 2D-BNNs. This can be an important point for effective release after uptake into the cells since intracellular compartments such as lysosomes are acidic. Furthermore, the conjugation of 2D-BNNs with folate and transferrin helped the effective targeting of cancer cells. The results indicated that the model drug, Dox, also accumulated in the nucleus of the cells as desired. Moreover, increased cytokine production could be attributed to the higher binding capacity and strong interaction of CpG oligonucleotides to 2D-BNNs. This evidence strongly suggested that 2D-BNNs are a potential candidate as an effective carrier for chemotherapy and immunostimulating drugs to improve their therapeutic efficiency and to reduce their side effects.

### 2D-BNNs in Biomaterial Applications

BN nanostructures are used to enhance the properties of composites due to their superior mechanical properties. Their dielectric properties, allowing a composite to maintain its electrical properties, can also be advantageous (Weng et al., 2014).

**Figure 7:**
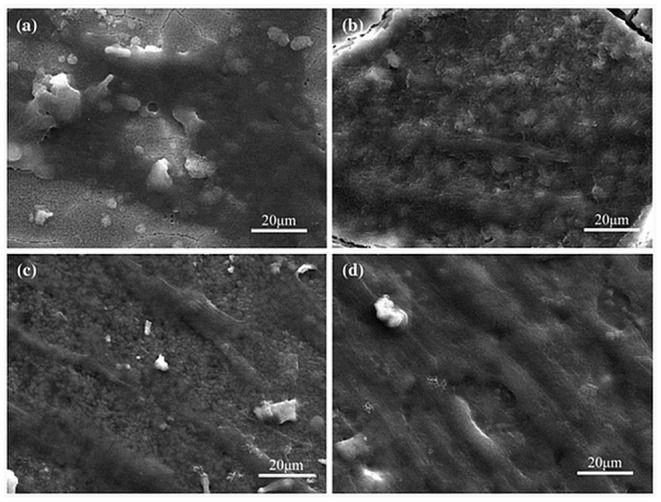
SEM images of BMSCs seeded scaffolds for **(a)** 1, **(b)** 3, **(c)** 5, and **(d)** 7 days. Reproduced with Permission from Shuai et al. (2015).

Farshid et al. used 0.2 wt% BNNTs and boron nitride nanoplatelets (BNNs) to obtain biodegradable poly(propylene fumarate) (PPF) nanocomposites for bone tissue engineering (Farshid et al., 2015). The results indicated that both nanomaterials significantly enhanced the mechanical strength of the polymer and the adsorption of the collagen I protein compared to the control PPF. Moreover, the cytocompatible polymeric nanocomposites showed improved extracellular matrix (ECM) deposition, cell attachment, and spreading, suggesting the use of the developed nanocomposites as bone grafts (Farshid et al., 2015). In another study, akermanite (AKM) scaffolds were fabricated using 0.5, 1, and 1.5 wt% 2D-BN nanosheets (Shuai et al., 2015). The significant increase in both the compressive strength and fracture toughness were found when the concentration of 2D-BNNs were increased from 0.5 to 1.0 wt%. Furthermore, the cell adhesion and proliferation studies on human osteosarcoma cells (MG63) and bone marrow stromal cells (BMSCs) indicated the cytocompatibility of the scaffolds for up to 7 days, as shown in Figure 7. The results indicated the potential of AKM scaffolds, including 2D-BNNs, in tissue engineering.

Gelatin electrospun mats (ESM) were fabricated using 0.1, 1, and 5 wt% hexagonal 2D-BNNs to improve mechanical properties (Nagarajan et al., 2017). The effects of cell adhesion, proliferation, biocompatibility, and osteoblast gene expression on osteosarcoma cell lines showed that the ESM are biocompatible and biodegradable, and that 2D-BNNs-reinforced gelatin scaffolds can be used for orthopedic applications thanks to the improved (3-fold) Young's modulus (Nagarajan et al., 2017). Jing et al. used 0.03, 0.06, 0.09, and 0.12 wt% hydroxylated 2D-BNNs (OH-BNNs) to fabricate poly(vinyl alcohol) (PVA) hydrogels (Jing et al., 2017). In this study, changing the content of OH-BNNs provided controllable reinforcements to both mechanical and thermal responses. Furthermore, the cytocompatibility of the hydrogels was tested using MTS and live/dead assays. The results suggested that OH-BNNs/PVA hydrogels have potential in tissue engineering, drug delivery, and artificial cartilages.

The composite of 2D-BNNs (using 1, 2, 3, 4, and 5 wt%) with poly(ether-ether-ketone) (PEEK) showed improved mechanical and thermo-mechanical properties since hBNs possess a high elastic modulus, excellent lubrication properties, and good thermal conductivity (Liu et al., 2015). In another study, 2D-BNNs-impregnated silane was added into magnesium (Mg) alloys to enhance the resistance of the corrosion for bioimplant applications (Al-Saadi et al., 2017). The results showed a 5-fold improvement in corrosion resistance in simulated human body fluid even after 96 h.

According to the results obtained from the biomaterials applications, it can be concluded that 2D-BNNs can be used as additives in nanocomposites or scaffold materials in tissue engineering and regenerative medicine since they enhance the mechanical and thermal properties of composite materials without interfering the electrical features of the polymer due to the high Young's modulus of BN nanostructures.

### Therapeutic Agents

Boron-including compounds such as calcium fructoborate, borax, boric acid (BA), boronic acid, and their esters were widely studied for their potential use in cancer treatment (Hegsted et al., 1991). These studies showed that boron-including compounds have remarkable inhibitory effects on cancer cell proliferation (Korkmaz et al., 2007; Scorei and Popa, 2010). BA, as a chemical form of boron in physiological conditions, has a tendency to make ester bonds with the hydroxyl groups of organic compounds (Van Duin et al., 1984). Therefore, hydroxyl groups that include structures such as carbohydrates are good candidates for the internalization of BA into their structure (Hausdorf et al., 1988). When the complexation is performed with structurally and functionally important carbohydrates, it manipulates the presence and activity of certain biological structures (Raven, 1980). These biomolecules include diadenosine phosphates (signal nucleotide) and S-adenosylmethionine used in methylation reactions of DNA, RNA, proteins, and phospholipids (Grill and Himmelbach, 1998; Ralston and Hunt, 2001). However, the systemic administration of soluble boron compounds frustrates the therapeutic effects of the structure owing to their short half-life in organisms.

The behavior of 2D-BNNs in biological media has a great importance since they may show their desired or undesired effects through their interaction with cellular compounds. For instance, 2D-BNNs, which are used as therapeutic agents, should be flexible enough to pass through barriers of reticuloendothelial system (RES) and should be stable enough to resist early degradation. In addition, the possible role of their degradation product should be clarified from therapeutic and adverse effect perspectives. However, it is not clear what the degradation products of BN-based nanomaterials might be. There is strong evidence that they will slowly degrade in biological media (Li et al., 2017; Sen et al., 2018). Based on *ab initio* calculations of several h-BN phases and c-BN phase nanostructures, the stability of both phases changed depending on the energy differences between the stacking geometries and inter-layer spacing, which can be altered with pressure or temperature (Mosuang and Lowther, 2002). As predicted from this study, our experimental findings strongly suggest that the crystallinity has an influence on the stability of hBNs in solution, and the degradation rate changed with the crystallinity (Sen et al., 2018). The question of the degradation products remains elusive at the moment. When the structure of BN nanomaterials is considered, it is logical to assume the products are either B or N compounds. From the B atom point of view, considering BA as one of the degradation products is realistic.

**Figure 8:**
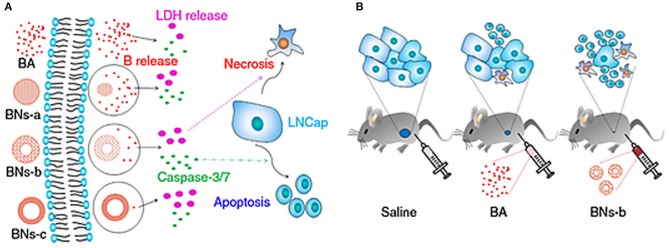
Effects of hollow 2D-BNNs on cellular and *in vivo* subcutaneously injected prostate cancer models. **(A)** BA or hollow 2D-BNNs with controlled B release resulting into different LDH release and caspase-3/7 activity in LNCaP prostate cancer, which is responsible for necrosis and apoptosis, respectively; **(B)** effects of saline, BA, and hollow BNNs-b spheres on mice pre-injected with LNCaP prostate cancer cells (Li et al., 2017).

In a study, three different kinds of hexagonal-based hollow 2D-BNNs, grouped depending on their degradation performance, were investigated as boron reservoirs against prostate cancer, as shown in Figure 8 (Li et al., 2017). The low crystallinity (BNNs-a), moderate crystallinity (BNNs-b), and high crystallinity (BNNs-c) effects of structures were evaluated on androgen-dependent LNCaP prostate cancer cells. Moreover, the chemical form of boron in physiological conditions, BA, was used as control owing to the degradation product of hollow 2D-BNNs. This study indicated that hollow BNNs-b spheres lead to a significantly higher fraction of apoptosis and necrosis rather than BA or with respect to the BNNs-a spheres with low crystallinity. Furthermore, hollow BNNs-b spheres and PTX drugs, commonly used drug for prostate cancer, were tested on LNCaP cell-injected male BALB/c-nu/nu mice models. The results indicated that hollow BNNs-b spheres showed more suppressive effects on tumors as compared to the PTX drugs. Besides, the hematological tests performed on hollow BNNs-b spheres injected in healthy C57/BL6 mice shows their biocompatible nature.

These progressive studies clarifying the underlying mechanism of biodegradation may provide tremendous opportunities to choose desired 2D-BNNs for targeted therapy. Moreover, in the light of the preliminary results on prostate cancer, the molecular mechanism of therapeutic efficiency should be further studied in detail to have an insight into their possible effects, not only on prostate cancer, but also on other cancer types.

### Pharmaceutical Agents

In pharmaceutical applications, a variety of lubrication agents has been studied to improve physical and chemical solid drug properties. The friction between the interfaces of the drug surface and the die wall of the inner surface of the tablets must be decreased to prevent the sticking of drug to the die while enhancing the softness and tensile strength of drug tablets. Therefore, appropriate lubricants should be used to improve the fluidity, filling properties, and efficiency of drug tables (Aoshima et al., 2005). In addition, the lubricants should be biocompatible, chemically inert, sustainable, and reproducible from batch to batch, and they should not cause adverse effects on the final dosage form (Miller and York, 1988). Considering the unique properties of 2D-BNNs, they are suitable nanostructures to be used as lubrication agents. Besides, their inertness also makes them suitable candidates for their use as lubricant agents (Wang et al., 2018).

The first study of hexagonal 2D-BNNs as lubricants was performed by Turkoglu et al. in 2005 (Turkoglu et al., 2005). The required lower punch force (LPEF) was tested on 2D-BNNs-including lubricants by comparing them with magnesium stearate (MGST), stearic acid (STAC), and glyceryl behenate (COMP). Based on the LPEF results, a better lubrication was obtained from 1% 2D-BNNs and MGST compared to the ones made from STAC and COMP. Moreover, the 2% MGST showed maximum performance in LPEF when it was mixed with 2D-BNNs that decreased LPEF to 50% as compared to only MGST-including ones (Turkoglu et al., 2005).

Later, several concentrations (0.5, 1.0, and 2.0 %) of STAC, COMP, hBN, and MGST lubricants were tested for their performance in LPEF applications (Ugurlu and Turkoglu, 2008). Tensile strengths of compacted tablets were measured by applying a diametrical load across the edge of tablets. The deformation mechanism of the tablets was also tested during compression. The study found that 2% of STAC and COMP did not decrease the LPEF as much as 0.5% of 2D-BNNs and MGST. 2D-BNNs and MGST were the most effective lubricants based on the LFEP-lubricant concentration profile. Moreover, the higher concentration of 2D-BNNs caused lower mechanical properties of tablets because of their hydrophobic character (Ugurlu and Turkoglu, 2008).

These studies showed that 2D-BNNs decreased LPEF in lubricants by improving the effects on disintegration time and tablet tensile strength, thus demonstrating their improved lubrication potential with respect to MGST. These results are encouraging for their use as lubricants in pharmaceutical applications.

### Cosmetic Applications

The cosmetic research to improve product properties is growing very fast. There are many materials utilized in cosmetic products (Arraudeau et al., 2003). However, they have a tendency to accumulate in furrows and wrinkles in the skin so that it further emphasizes the wrinkles instead of showing smoother skin by hiding the wrinkles. Moreover, spherical powders, including silica, polyethylene, and polymethylmethacrylate (PMMA), are utilized as skin shiners and wrinkles hiders by blurring the appearance of skin through the use of the structure's light-scattering properties (Macchio et al., 1988; Lemperle et al., 1998; Pölloth, 2005).

The earliest use of BNs in cosmetics was reported as being a slip modifier, but it was not indicated which crystal form was used (Gottschalck and Breslawec, 2012). However, it is assumed that it is the hBN form because the pursued functionality in cosmetics corresponds to the properties of this crystal form, and BN used in cosmetics is not listed as a nanomaterial. In addition, it has been reported that the average particle size of hBNs used in cosmetics ranges from 1 to 47 mm depending on their trade names and grade (Monice et al., 2015). The assumption made by the industry was, perhaps, not due to a lack of awareness of their unique properties as nanomaterials but just because of their lubricating properties.

Based on the US Food and Drug Administration (FDA), hBNs are used in more than 650 cosmetic formulations, including lipstick, eye shadows, foundation agent, blush, shampoo, and hair conditioner, as transparent raw fillers. They enhance skin appearance by not only blurring lines and wrinkles but also by providing coverage of the age spots, blemishes, and discolorations (Butts et al., 2007). 2D-BNNs fillers are also used in cosmetic products combined with other materials, including spherical silica, PMMA, and titanium. Apart from their filling properties, it was surprising to observe that they created an illusion of smoothness on the skin by hiding the wrinkles, thereby making them promising agents in cosmetic applications (Newman et al., 2015). Moreover, the 2D-BNNs could be coated with special amino acids and mineral oils that improve the ingredients of the cosmetic product (Koshida et al., 2019).

The use of 2D-BNNs in cosmetic application is now welcomed due to their biocompatibility and transparency, which is highly desired in white formulations. Furthermore, 2D-BNNs could be easily dispersed in oily cosmetic formulations due to their hydrophobicity, and this consequently improved the formulation's homogeneity. As their properties are better understood, their use in more cosmetic products will appear.

## Conclusion and Future Perspectives

As highlighted in this review, 2D-BNNs are considered good candidates for a wide range of biomedical and bio-related applications, such as in drug and gene delivery, biomaterials, pharmaceutics, and cosmetics, due to their excellent physicochemical properties. Considering the synthesis of 2D-BNNs, it is important to choose appropriate precursors since the structure, crystallinity, and purity of the final product strongly depends on the B precursor and catalyst. The other experimental parameters, including temperature and pressure, are important for crystallinity and product quality. Not being able to synthesize at large-scale and high-temperature requirements are still bottlenecks for their widespread use. It is clear that more research efforts should be devoted to the development of synthesis procedures at lower temperatures. The high chemical stability and hydrophobicity of the 2D-BNNs result into their poor dispersibility in aqueous media, which hinders the reliable assessment of their effects on living systems. Significant effort has therefore been dedicated to their functionalization for dispersions in physiological solutions and their interactions with other biomaterials. Moreover, biocompatibility investigations of 2D-BNNs have been performed to clarify their acceptability in biomedical applications. Although it is found that 2D-BNNs are non-toxic in *in vitro* studies, depending on the cell types, concentrations, and lateral size dimensions, *in vivo* investigations should be further performed in future studies in order to reach more concrete conclusions.

The possibility of functionalizing 2D-BNNs though their –OH or –NH_2_ groups with a variety of molecular structures makes them suitable carriers. The short half-life of boron compounds in biological systems—caused by their fast metabolism—is a significant problem in therapeutic applications. Using 2D-BNNs in place of these compounds might be a good option in order to maintain the sustained released of boron into the biological system. The superior mechanical properties of 2D-BNNs are utilized to strengthen polymeric structures. The dispersion problem of highly hydrophobic 2D-BNNs in polymer mixtures could be overcome with their surface modifications with a suitable molecular structure. Using 2D-BNNs as additives in cosmetic products has already shown improvements in product quality and the functionalization of these novel materials to match their chemical compatibility in formulations can add another dimension for their use in cosmetics. Although the findings indicate that these nanomaterials are quite promising, researchers should put more effort into better understanding the role and behavior of BNs in a biological matrix to be able use them in biomedical applications. Finally, the establishment of a clear structure–function relationship is necessary to fully benefit from the unique properties of these novel materials in a wide range of applications.

## Author Contributions

ME and ÖS wrote the manuscript. IT organized the references. MÇ guided the study and revised the manuscript.

### Conflict of Interest

The authors declare that the research was conducted in the absence of any commercial or financial relationships that could be construed as a potential conflict of interest.
